# Spirituality and religiosity as predictors of subjective wellbeing in older Mexican adults

**DOI:** 10.3389/fpsyg.2025.1663757

**Published:** 2025-11-13

**Authors:** Alejandro González-González, Patricia Andrade Palos, Rosa Estela García-Chanes, María Enriqueta Sánchez Hernández, Diana Betancourt Ocampo

**Affiliations:** 1 Faculty of Psychology, Universidad Anáhuac México, Mexico City, Mexico; 2 Faculty of Psychology, Universidad Nacional Autónoma de México, Mexico City, Mexico; 3 Research Department, Instituto Nacional de Geriatría, Mexico City, Mexico

**Keywords:** religion, health, spiritual, aging, wellbeing

## Abstract

**Objective:**

This study analyzed the effect of spirituality and religiosity on subjective wellbeing in Mexican older adults, controlling some sociodemographic variables (e.g., sex, age, schooling, who they live with, monthly income) and health variables (e.g., health perception, difficulties in carrying out activities, presence of medical conditions, depression).

**Methods:**

A non-probabilistic sample of 923 older adults ( $\bar{x}$ *=* 74.49, SD *=* 7.46) was obtained, 74.8% were women and 25.2% men. The EBS-8 subjective wellbeing scale was used to assess the dependent variable. To assess religiosity, the Religious Involvement dimension of ASPIRES was, and to assess spirituality it was used the dimension of Importance of Spiritual Beliefs of the Spiritual Questionnaire, both dimensions in their Spanish-adapted versions. Was used a questionnaire with 16 closed-option questions which evaluate self-perception of health, self-reported difficulty in performing activities of daily living and the presence of medical conditions. The scale adapted to the Mexican population of the Yesavage Geriatric Depression Scale (GDS) was used.

**Results:**

The results showed that spirituality and living with a partner have positive and significant effects on subjective wellbeing, while depression and difficulties in performing activities of daily living reduce it, this regression model explained 17% of the variability in subjective wellbeing.

**Conclusion:**

In the present study, it was observed that in each of the models tested, spirituality played an important role as a predictor of subjective wellbeing, and even remained stable when demographic (sex, age, schooling, who they live with) and health (health perception, difficulties in carrying out activities, depression) variables were included.

## 1 Introduction

One of the fundamental premises in all countries is being able to guarantee a healthy life and promote the wellbeing of all people, regardless of their condition or age, which will contribute to the construction of prosperous and healthy societies, so it is important to consider what the outlook is in relation to population changes, in particular, with the increase in the number of older adults (OA) and their needs, projections for 2050 indicate that one out of every six people in the world will be over 65 years of age (United Nations, 2022), which represents an increase from 10% in 2022 to 16% in 2050.

In Mexico, as in other countries around the world, there is an accelerated increase in the number and proportion of OA. By 2030, one in six people worldwide is expected to be 60 years of age or older and it is expected that by 2050 this proportion will double, which represents one of the greatest challenges in terms of public health and the social conditions in which these OA will live (Organización Mundial de la Salud, 2025). In addition, the way in which people are aging is not observed in the best conditions, considering the prevalence of chronic diseases, diet and lifestyle habits, as well as persistent social and gender inequalities. In this regard, the Inter-American Development Bank indicates that chronic diseases are highly prevalent among OA in Latin America and increase with age, as well as being a major cause of mortality and morbidity (Aranco et al., 2022). According to data provided by Mexican Health and Aging Study (MHAS) 2021, 22.7% have diabetes, 38.5% have hypertension and 54.6% report having a regular state of health; in relation to their mental health and emotional wellbeing, 59.4% indicated that they have a bad to regular quality of memory, while 25.3% had depressive symptoms. Although these data reflect some problems, their evaluation of life satisfaction is high, since nine out of 10 OA reported being satisfied (Instituto Nacional de Estadística y Geografía, 2021).

Other studies (García-Chanes et al., 2024; López-Ortega et al., 2016) show that the psychosocial wellbeing of Mexican adults is associated with a positive perception of the economic situation, the absence of depressive symptoms, and social participation rather than with the presence of comorbidity. In this same vein, studies in Mexico (Instituto Nacional de Estadística y Geografía, 2021) indicate that among the older adult population, men have higher life satisfaction scores than women. Reyna-Barajas et al. (2025) found that older adult men report a better quality of life in terms of physical performance and perception of wellbeing, while women have a higher prevalence of chronic diseases. In addition, the authors point out that men and women may experience aging differently due to biological, psychological, social, and cultural factors, which can directly affect quality of life in old age.

Globally, the World Health Organization (Organización Mundial de la Salud, 2022) proposed the Healthy Ageing framework that focuses on developing and maintaining functional ability in older people, understood as the basis for achieving wellbeing in later life. In context, the Decade of Healthy Ageing 2020–2030 is an initiative aimed at improving the lives of older people, their families and communities and promoting healthy aging. To achieve this, collaboration between governments, civil society, international organizations, academia, the media and the private sector is encouraged.

In particular, subjective wellbeing (SW) refers to the evaluation that people make of their own wellbeing and is part of people’s quality of life. It arises from the importance of considering not only economic and social aspects to define people’s quality of life, since people react differently to the same circumstances and evaluate their conditions based on their expectations, values and experiences (Diener, 1984; Diener and Suh, 1997; Diener et al., 1999). SW is conceptualized as hedonic wellbeing because it emphasizes pleasure and satisfaction (Tov et al., 2022) and contemplates two dimensions: the cognitive one that is generated through satisfaction with oneself, family and society, and the affective one that focuses more on affections, emotions, feelings and positive and negative moods (Diener, 1984; Arévalo-Avecillas et al., 2019; Flores-Kanter et al., 2018).

Triyuliasari et al. (2024) made a systematic review of research that analyzes different factors that influence the SW of OA and conclude that there is empirical evidence that sociodemographic aspects (educational, marital and economic status), social activities (voluntary activities, religious), physiological (health and sleep quality) and psychological factors (family support, spirituality and religiosity, self-efficacy, internet use) all have an impact on the SW of OA. Regarding sociodemographic variables such as gender, age and economic income, the results are not consistent and perhaps this is due to the different measures of quality of life and wellbeing (Batz and Tay, 2018; Gobbens and Remmen, 2019; Lansford, 2018), it seems that life experiences and decisions play a more important role, as well as environmental variables.

In terms of health-related variables, it has been shown that physical health is positively related to SW (Cross et al., 2018), and in the case of OA, an important aspect is independence and autonomy, so maintaining the ability to perform daily activities and make personal decisions that are linked to greater satisfaction and wellbeing with life are fundamental in their personal situation (Jitdorn et al., 2021; Ladusingh and Ngangbam, 2016). However, given that physical health declines with age, one might expect SW to decline, but scientific evidence points to higher SW at older ages, suggesting that changes related to family, work, social networks, and physical capacity associated with aging individuals should rather be studied (Stone et al., 2020).

Some aspects of mental health related to SW and life satisfaction have also been studied (Hashmi et al., 2018; Lombardo et al., 2018; Ngamaba et al., 2017), but some measures of mental health have been done through self-reports, which consistently correlate; however, the relationship diminishes when objective measures are included (Diener et al., 2018). Depression is one of the most studied variables and has been found to decrease the SW of OA (Li et al., 2023; Lukaschek et al., 2017; Soósová et al., 2021).

Among the factors that some authors have found as part of the wellbeing of OA is religiosity and spirituality, referring to the fact that the practices of meditation, orientation, prayers and church events generate certain feelings of satisfaction, self-improvement and security that lead them to become stronger, in addition to providing them with a sense of life (Gallardo-Peralta and Sánchez-Moreno, 2020; Zapata, 2017). The experience of religiosity and spirituality can be shared collectively, but it is recognized that these experiences are unique and unrepeatable for each person who lives them, and particularly in the case of OA (Casanova et al., 2024; Guerrero-Castañeda et al., 2021; Tabatabaei and Ebrahimi, 2023; Watkins et al., 2022). Unlike religiosity, spirituality does not deny or affirm a divine origin, but refers to a search for meaning in life, which may or may not be related to belief in God or other equivalent cultural dimensions (De Freitas et al., 2022).

Dominguez et al. (2024) analyzed the evidence of the relationship between spirituality/religiosity with longevity and concluded that numerous epidemiological studies show that high levels of spirituality/religiosity in its different forms are associated with lower mortality rates. Likewise, Malone and Dadswell (2018) and Papadopoulos (2020) note the importance of religion and spirituality in positive aging as they can be a source of strength, comfort, and hope, as well as being a sense of community and belonging. Litalien et al. (2022) concluded that religiosity/spirituality have influence on healthy behaviors and wellbeing, but more studies by sex are needed.

Zimmer et al. (2016) indicate that although there is evidence that spirituality/religiosity is associated with long life and greater physical and mental health, the two constructs need to be investigated separately. While the two concepts are related and most people who consider themselves religious also consider themselves spiritual, not all people who are spiritual consider themselves religious. Religion is generally associated with beliefs, practices, and rituals, whereas spirituality is more difficult to define, as it means different things to different individuals in different places. The authors point out that it is easier to quantify religiosity than spirituality and some studies show that spirituality has greater weight than religiosity in the SW of OA (Gallardo-Peralta and Sánchez-Moreno, 2020; Saldías-Ortega and Moyano-Díaz, 2023).

Büssing (2018) did a systematic review of instruments measuring spirituality/religiosity in clinical settings and proposes like Zimmer et al. (2016) that they are two related constructs but with different definitions, so they should be measured as two independent constructs. He further notes that religiosity has generally been measured as adherence to beliefs, doctrines, ethics, rituals, texts, and practices associated with high power and spirituality is defined as a set of internal feelings and experiences through which a person seeks meaning and purpose, as well as relationship to self, family, society, nature, and the meaning of the sacred. Spirituality is something more subjective and different aspects of spirituality have been measured, such as levels of spirituality, spiritual wellbeing, spiritual distress, spiritual needs. In terms of religiosity, religious beliefs and practices and level of religiosity are generally measured.

Regarding religiosity, the findings indicate that it has a positive influence on the wellbeing of OA because its practices offer better ways of coping with the challenges of this stage, as well as preparing them to fulfill their life mission, although this is often uncertain (Coelho-Júnior et al., 2022; Soriano et al., 2021). In addition to this, it has been found that active involvement in a religious community, either by praying or doing work dedicated to others, generates greater feelings of belonging and usefulness; specifically in women, religiosity allows them to live together at a time when most of their time is spent at home (Tabatabaei and Ebrahimi, 2023; Vicente et al., 2018). Some studies show that maintaining social interactions, which occur through religious practices, contribute significantly to their sense of belonging and life satisfaction, which results in their overall wellbeing (Jitdorn et al., 2021; Cramm and Nieboer, 2015; Dorji et al., 2019; Du et al., 2023; Paul et al., 2024).

On the other hand, it has been found that spirituality has a positive influence on OA (Soósová et al., 2021) since it helps them to achieve a greater connection with themselves and with others, as well as allowing them to have a positive attitude towards the changes and stress that come with grief and challenging situations that they go through during this stage (Can Oz et al., 2022). Spirituality has also been considered a protective factor for OA as it helps them cope with physical, psychological and social changes, in addition to providing them with security, stability and meaning in their lives (Pérez et al., 2022). Neville et al. (2024) found that higher spirituality, independent of religiosity, was independently associated with good self-rated health in geriatric outpatients. Being able to enhance spirituality seems to represent one of the relevant intervention strategies in the prevention and treatment of depressive symptoms and improvement of wellbeing in OA (Soósová et al., 2021).

In general, religious practices and spirituality have been considered an important resource in OA, as they help them cope with stress and in several studies are associated with less depression and anxiety and greater hope. Religious/spiritual involvement has even been shown to be a powerful resource for psychiatric patients, as it is related to mental (Koenig and Al Zaben, 2021; Koenig et al., 2020) and physical health (Abu et al., 2018). However, in a review of studies on physical and mental health and religiosity/spirituality, Lucchetti et al. (2021) point out that the results are not conclusive, as some authors have not found significant relationships.

Hence, it is worthwhile to carry out studies with populations characterized by a series of unfavorable health conditions (for example, the presence of chronic diseases), as well as living conditions that represent some disadvantages to face aging (Aranco et al., 2022), and therefore it would be expected that their wellbeing would be affected. This is the case in Latin America, where OA live in conditions characterized by a series of structural deficiencies, including access to social and health services, which contributes to the transition through this stage of old age involving complications and obstacles that affect their perception of health and possibilities for improvement. In addition, a high percentage of OA continue to work and sometimes cannot count on a decent retirement, it is here where spirituality and religiosity would become relevant to contribute to a better wellbeing. All this would offer the possibility of incorporating these variables as promoters of wellbeing in contexts such as those experienced in Latin American countries where there are numerous disadvantages that imply a greater challenge for health and social care systems.

The aim of this study was to analyze the effect of spirituality and religiosity on SW in Mexican OA controlling for sociodemographic (e.g., sex, age, schooling, who they live with) and health (e.g., health perception, difficulties in carrying out activities, depression) variables. The central hypothesis of this research is that spirituality and religiosity are predictor variables of SW in OA independently of some sociodemographic and health characteristics.

## 2 Materials and methods

### 2.1 Participants

A non-probabilistic sample of OA residing in Mexico City was used, consisting of 923 OA between 60 and 100 years old (*M* = 74.49, *SD* = 7.46). The participants had some kind of contact with the Instituto para el Envejecimiento Digno (INED) in Mexico City, either because they attended spaces where they could regularly socialize with other OA, share experiences, and stay physically and mentally active. They could also be OA who were visited at home by staff from the institute to follow up on a particular need (e.g., physical, psychological, legal, etc.). It is important to note that the care provided by INED focuses mainly on older adults living in neighborhoods identified as having a low or very low Social Development Index.

### 2.2 Measures

#### 2.2.1 Dependent variable

The Subjective Wellbeing Scale (EBS-8, acronym in Spanish) by Calleja and Mason (2020) was used, which measures life satisfaction (e.g., Likes his life, He is satisfied with his life) and positive affect (e.g., He is a happy person, Your life brings you joy), and consists of eight items in Likert-type format with four response options (from Disagree to Totally Agree), it is an instrument that was developed for the Mexican population and has been applied in adolescents, adults and OA, where it has shown empirical evidence of validity and reliability (*α* = 0.95).

#### 2.2.2 Independent variables

The dimension of Religious Involvement was used, which is part of the main dimension of Religious Feelings of the Assessment Scale of Spirituality and Religious Feelings (ASPIRES) Scale, originally developed by Piedmont (2014) and validated in Spanish by Simkin (2017). The dimension used consists of eight items (*α* = 0.85) in a Likert-type format with 5–7 types of responses, and reflects how actively involved a person is in performing various religious rituals and activities (e.g., How often do you pray? How often do you attend mass or ceremonies specific to your religion?). It should be noted that this instrument does not have psychometric evidence in Mexico, which is why, as part of this study, its performance will be analyzed in the sample studied.

To evaluate spirituality, the dimension of importance of spiritual beliefs (*α* = 0.91) of the Spiritual Questionnaire by Parsian and Dunning (2009) was used, which consists of four items (e.g., Spirituality helps me define the goals I set in my life, Spirituality helps me decide who I am) in a Likert-type format with six responses ranging from never to always. The Spiritual Questionnaire did not report evidence of validity and reliability in the Mexican sample, which is why this study will analyze its performance in the sample studied. In addition, the scale adapted to the Mexican population (Salgado et al., 2024; Yesavage et al., 1983) of the Yesavage Geriatric Depression Scale (GDS) was used, consisting of 14 items (e.g., Do you feel happy most of the time? Have you dropped many of your activities and interests?) in dichotomous format (yes/no), which obtained adequate psychometric values in a sample of Mexican OA (*α* = 0.94).

A questionnaire with 16 closed-option questions was included based on items included in the Mexican Health and Aging Study (MHAS) 2021 (Instituto Nacional de Estadística y Geografía, 2022) and validated in the Mexican population, which evaluate: self-perception of health (excellent, very good, good, fair, or bad), the presence of medical conditions (e.g., diabetes, hypertension, arthritis) and, self-reported difficulty in performing activities of daily living (BAVD) was included using a dichotomous variable categorized as 0 (no difficulty) and ≥1 for difficulty in performing one or more of the following activities: walking, bathing, eating, lying down and using the toilet. In addition, a sociodemographic data questionnaire was included, asking for sex, age, education, marital status, who they lived with, current occupation and monthly income.

### 2.3 Procedure

The study was supported by the Secretaría de Bienestar e Igualdad Social of México City, through the Instituto para el Envejecimiento Digno (INED). Part of the work included a training process for the staff who participated in the study with the purpose of making them familiar with and learning how to improve the instruments that would be applied. After the training, the INED staff went to the home of each OA, where the objective and scope of the study was explained to them with the intention of obtaining their consent and voluntary participation. Likewise, they were informed that the information collected was confidential and would be used only as part of the research. The Mini-Cog (Borson et al., 2003) was applied to OA who initially agreed to participate, to confirm they had the cognitive capacity to answer the instruments. The criterion used was that if the older adult remembered two or three words, the rest of the instruments were applied in the form of an individual interview. The OA did not receive any incentive for participating in the study.

### 2.4 Data analysis

The analysis was conducted in the following sequential steps. First, a descriptive analysis was performed to examine the characteristics of the sample. Next, confirmatory factor analyses (CFA) were then performed for each of the scales used and the following values were considered as adequate fit indices (Hu and Bentler, 1999): the *χ*^2^/df ratio (CMIN/DF) with a value <3, the CFI (Comparative Fit Index) and TLI (Tucker–Lewis Index) indices with values >0.90; and RMSEA (Root Mean Square of Approximation) with a value <0.08. Reliability was calculated using Cronbach’s alpha. The means and standard deviations were calculated for the scales used.

Subsequently, the average scores of the SW scale were compared across various characteristics and health conditions using descriptive analysis. Depending on the type of variable, analysis of variance (ANOVA) tests and independent means tests were applied. Additionally, a correlation analysis was conducted to assess the strength and significance of the association between spirituality and SW scales and several quantitative covariates. These covariates included the dimension of religious involvement, age, self-reported health, depression scale and the number of basic activities affected (BAVD), with the Spearman test employed for this analysis.

To examine the role of spirituality in SW (dependent variable), three linear regression models were estimated. Model 0 focused solely on the effect of the spirituality scale on SW. In Model 1, sociodemographic variables were included to assess any changes in the coefficient associated with spirituality. Finally, Model 2 incorporated both sociodemographic variables and health conditions to provide a more comprehensive analysis. Statistical analyses were performed with STATA version 14.0 and Jamovi version 1.6.23.0.

## 3 Results

Regarding the sociodemographic characteristics of the participants, the majority were women (74.8%), with a mean age of 74.5 years. Half of the participants attained at least a mid-level education, with 30.2% having completed high school and 20.6% holding an undergraduate degree or higher. Additionally, 41.0% were married or in a partnership, while 16.3% are single. Only 10.4% were currently employed, 29.6% were retired, and 55.2% were homemakers. Furthermore, 15.7% live alone, 33.9% live with a partner, and half reside with other family members (Table 1). Regarding health conditions, the highest percentage reported good health, while one-third indicated that they had at least one medical condition, furthermore, 22.9% indicated difficulty performing at least one basic activity of daily living (Table 1).

**Table 1:** Sociodemographic and health characteristics of the participants.

Characteristics	*n*	%
Sex		
Men	233	25.2
Women	690	74.8
Age group		
60–69	251	27.2
70–79	449	48.7
80+	223	24.2
Schooling		
Without schooling	113	12.4
Elemental	337	36.9
Middle/high-school	276	30.2
Undergraduate and more	188	20.6
Marital situation		
Single	150	16.3
Married/cohabitation	378	41.0
Ever married (separated, divorced or widowed)	395	42.8
Current occupation		
Employees	95	10.4
Unemployment	45	4.9
Retired	271	29.6
Homemakers	506	55.2
Monthly income category^*^		
Does not know	92	10.1
Up to $1,499	89	9.8
From $1,500 to $2,999	201	22.0
From $3,000 to $4,999	271	29.7
From $5,000 to $7,999	160	17.5
$8,000 or more	99	10.9
People you live with		
Alone	144	15.7
With partner	310	33.9
With other people or family	461	50.4
Self-rated heath		
Excellent	28	3.1
Very good	205	22.4
Good	350	38.4
Regular	287	31.4
Bad	43	4.7
Comorbilities		
0	269	29.1
1	323	35.0
2	232	25.1
3+	99	10.7
BADL		
0	712	77.1
1+	211	22.9

Basic Activities of Daily Living (BADL): difficulty walking, bathing, eating, lying down and using the toilet. ^*^Amount in Mexican pesos.

Table 2 shows the results regarding the CFA, Cronbach’s alpha, mean and standard deviation for the tools used in this study. As can be seen, in general the indices were adequate for all instruments. The results showed that for most of the instruments an adequate fit was found for CFA, in addition to obtaining adequate Cronbach’s alpha values. Only for religious involvement three items had to be eliminated (r1, r2 and r8) based on the modification indices for the instrument to show an adequate fit.

**Table 2:** Evidence of reliability and validity of the instruments used.

Measure	*X*^2^ (*gl*)	CMIN	TLI	CFI	RMSEA (IC)	*α*	$\bar{x}$	SD
EBS-8	134.86 (20)	6.74	1	1	0.065 (0.055–0.075)	0.970	4.77	1.18
GDS	3548.39 (91)	38.99	0.887	0.905	0.059 (0.054–0.065)	0.765	2.41	2.16
Importance of spiritual beliefs	8.01 (2)	4.00	0.992	0.997	0.057 (0.020–0.101)	0.898	4.34	1.47
Religious involvement	21.2 (5)	4.2	0.977	0.989	0.059 (0.034–0.086)	0.801	3.78	0.82

Score range: Importance of spiritual beliefs and EBS-8 from 1 to 6; Religious Involvement from 1 to 5; GDS from 0 to 13.

When comparing the average scores of the SW scale across various characteristics and conditions (Table 3), the results reveal distinct patterns. No significant differences were found based on sociodemographic characteristics such as gender, age, education level, marital status, or household arrangement. However, individuals with better self-perceived health and no functional limitations reported higher SW scores. In addition, OA with a greater number of health conditions had lower SW.

**Table 3:** Mean subjective wellbeing index by different characteristics and health conditions.

Variables	Subjective wellbeing	
	$\bar{x}$	SD
Total	4.8	1.2
Sex		
Men	4.7	1.1
Women	4.8	1.2
Age group		
60–69	4.8	1.2
70–79	4.8	1.2
80+	4.7	1.3
Schooling		
Without schooling	4.6	1.2
Elemental	4.7	1.2
Middle/high-school	4.8	1.1
Undergraduate and more	4.9	1.2
Marital situation		
Single	4.7	1.3
Married/cohabitation	4.8	1.1
Ever married (separated, divorced or widowed)	4.7	1.2
People you live with		
Alone	4.8	1.2
With partner	4.9	1.1
With other people or family	4.7	1.2
Self-rated heath		
Excellent	5.1	0.2^***^
Very good	4.9	0.1^***^
Good	4.7	0.0^***^
Regular	4.6	0.0^***^
Bad	4.3	0.1^***^
Comorbilities		
0	4.7	1.1^***^
1	4.9	1.2^***^
2	4.8	1.2^***^
3+	4.5	1.2^***^
BAVD		
0	4.9	1.1^***^
1+	4.3	1.3^***^

^***^p < 0.05. *t*-test and ANOVA as appropriate. BAVD, Basic Activities of Daily living.

In terms of correlations (Table 4), SW shows a positive association with spirituality, religiosity, and self-reported health. On the other hand, an increase in SW is related to a significant decrease in depression and the number of basic activities affected. Similarly, as spirituality increases, religiosity, and SW also increase. However, in an inverse relationship, the associations of spirituality with depression and are weaker compared to those observed with SW. It is important to note that no association was found between SW and age.

**Table 4:** Correlation of variables.

Variables	1.	2.	3.	4.	5.	6.	7.	8.
1. Subjective wellbeing	–							
2. Spirituality	0.30^*^	–						
3. Religiosity	0.21^*^	0.43^*^	–					
4. Age	−0.01	0.04	0.02	–				
5. Self-reported health	0.14^*^	0.03	0.01	−0.03	–			
6. Depression	−0.39^*^	−0.19^*^	−0.22^*^	0.06^*^	−0.27^*^	–		
7. Number of BAVDs affected	−0.19^*^	−0.05	−0.03	0.12^*^	−0.29^*^	0.26^*^	–	
8. Number of comorbilities	−0.03	0.02	0.08^*^	0.17^*^	−0.24^*^	0.18^*^	0.24^*^	–

^*^p < 0.05. BAVD, Basic Activities of Daily living.

Table 5 presents the estimates from the linear regression models analyzing the effects of spirituality, religiosity, sociodemographic characteristics, and health conditions (physical and mental) on the SW index. In Model 0, SW increases significantly when spirituality enters the equation. When sociodemographic variables are added in Model 1, the significant effect of spirituality persists, although it slightly decreases and a significant effect of schooling is presented, where SW increases in OA with higher education. In Model 2, which incorporates health conditions, there were significant effects of spirituality and depression. Among the health variables, the results showed that difficulties in basic activities significantly reduce SW. In this final model, the R-squared value rises to 0.174, indicating that approximately 17% of the variability in SW is explained by the variables included in the model.

**Table 5:** Results of linear regression models with subjective wellbeing as the dependent variable.

Variables	Model 0		Model 1		Model 2	
	Spirituality		+Sociodemographic variable		Sociodemographic + health variables		
Coef.	*P* > |*t*|	Coef.	*P* > |*t*|	Coef.	*P* > |*t*|
Age			0.002	0.274	0.004	0.068
Sex						
Men (ref.)						
Women			0.000	0.991	0.022	0.574
Schooling						
Without schooling (ref.)						
Elemental			0.027	0.604	0.003	0.940
Middle / High-school			0.053	0.338	-0.002	0.970
Undergraduate and more			0.123	**0.033**	0.048	0.393
People you live with						
Alone (ref.)						
With partner			0.097	0.051	0.091	0.059
With other people or family			-0.011	0.819	-0.010	0.816
Depression					-0.059	**0.001**
Spirituality	0.09	**0.000**	0.081	**0.000**	0.066	**0.001**
Religiosity			0.042	0.063	0.003	0.879
Self -Rated Heath						
Bad (ref.)						
Regular					-0.052	0.493
Good					-0.083	0.285
Very good					-0.084	0.305
Excellent					-0.022	0.848
BAVD					-0.099	**0.015**
Constant	0.13	0.011	−0.247	0.221	0.018	0.926
*R^2^*	0.0716		0.0844		0.174	

BAVD, Basic Activities of Daily living. Bold values indicate *p* < 0.05.

## 4 Discussion

The central objective of this research was to analyze the importance of spirituality and religiosity in the SW of OA, controlling demographic and health variables, for which three models were tested, in the third model that explains 17% of the variance of SW it was found that spirituality, while depression and difficulties in daily life reduce SW.

The results allow affirming that spirituality plays an important role in the SW of OA, which coincides with the results of other authors (Soósová et al., 2021; Litalien et al., 2022; Can Oz et al., 2022; Pérez et al., 2022; Koenig and Al Zaben, 2021; Koenig et al., 2020) who affirm that spirituality can be a protective factor for OA as it helps them to cope with physical, psychological and social changes, in addition to providing them with some security, stability and sense of life. It also corroborates the importance of physical and mental health in the SW of OA as has been raised in several studies (Cross et al., 2018; Jitdorn et al., 2021; Ladusingh and Ngangbam, 2016; Hashmi et al., 2018; Lombardo et al., 2018), since physical health is related to functionality and autonomy, and for its part, taking care of mental health enables adequate management of the situations that arise in daily life, as well as adequate stress management.

It is important to highlight that in this study no significant differences were found in SW by sociodemographic variables, which contradicts the results of other studies (Lukaschek et al., 2017; Zhang and Sun, 2024), where it has been pointed out that there are differences, mainly in sex and age, where in the latter, there is not necessarily a decrease in SW as age advances, although on the other hand, differences are confirmed by health variables, such as self-perceived health and difficulties in daily activities reported by the people (Cross et al., 2018; Jitdorn et al., 2021), although it would be convenient for the case of the assessment of health conditions, to have objective data to corroborate these findings, beyond just assessing the self-perception of health status.

The correlation analysis showed relationships in the expected direction, the greater the spirituality, religiosity and self-perceived health, the greater the SW. This could confirm that these variables promote positive aspects in the OA, in this sense it can be mentioned that spirituality and religiosity have been shown to be variables that favor feelings of hope, as well as an adequate meaning of their own life and a greater belonging to their community and/or social groups. In the case of self-perceived health, this can be linked to an adequate SW as a result of the perception of their condition and their functionality, which is congruent with the correlation found between self-perceived health and the number of difficulties in carrying out activities of daily living, where the better the perception of health, the fewer the number of difficulties; in addition, the number of difficulties was negatively associated with SW.

The highest SW correlations were obtained with depression and spirituality, which is congruent and confirms that depression decreases the SW of OA (Li et al., 2023; Lukaschek et al., 2017; Soósová et al., 2021) by negatively impacting the lives of OA. Regarding the role of spirituality, it is observed to increase SW (Soósová et al., 2021; Can Oz et al., 2022; Abu et al., 2018) by fostering a sense of connection with themselves and others, as well as a sense of hope, transcendence, and wisdom in the face of the conditions that aging sometimes represents. An important finding was that spirituality is related to lower depressive symptomatology, which could represent a feasible intervention strategy in the prevention and treatment of depressive symptoms (Soósová et al., 2021; Gallardo-Peralta and Sánchez-Moreno, 2020), particularly as a coping mechanism in the face of adverse situations in the lives of OA, by mobilizing their internal resources to promote hope, a sense of belonging and connection with themselves, with others and with nature.

Another interesting result is that spirituality showed a stronger correlation with SW than religiosity, which confirms the results of other studies (Saldías-Ortega and Moyano-Díaz, 2023) and underlines the importance of measuring these two constructs independently as several authors have proposed (Zimmer et al., 2016; Büssing, 2018), in order to be able to evaluate the effect of each one, because although the two constructs are related, they are different, conceptually and operationally, otherwise, it will be assumed that both play the same role, and could even hinder the ability to differentiate possible additive effects between these two constructs.

In the present study, it was observed that in each of the models tested, spirituality played an important role as a predictor of SW, and even remained stable when the demographic variables of sex, age, schooling, and who the OA lives with were incorporated, maintaining itself even with the inclusion of variables related to the health conditions reported by the OA, and although it was found that, in particular depression and difficulties in performing activities of daily living negatively affected SW, spirituality continues to be a variable that favors SW in the sample analyzed.

These results contribute to the analysis of possible strategies that can promote healthy aging, such as spirituality, which showed a positive relationship with the subjective wellbeing of OA and a negative relationship with depression. Therefore, it would be important to take into account that, when developing interventions that promote the wellbeing of older adults, spirituality should be included as a central element, incorporating activities that promote internalization and mindfulness into interventions, such as meditation, mindfulness, reflection and meaning activities such as writing or reading that promote reflection and self-awareness, activities involving interaction with people and groups that promote helping others and, therefore, foster transcendence and purpose in life, as well as the inclusion of outdoor activities that bring older adults into contact with nature and other living beings.

One of the limitations of this study was that there were no objective measures to assess health status, so it would be advisable for future studies to use a more accurate indicator and avoid any kind of bias. In addition, this study had a non-probabilistic sample and most of the participants were women, so it is recommended that these results be confirmed in other samples of OA. Another major challenge is the measurement of spirituality, which, especially in older adults, could be confused with religiosity, particularly in populations that have deeply rooted religious practices as part of their history and culture. In spite of this, the present research provided elements on the role of spirituality and religiosity in a sample characterized by certain unfavorable health and life conditions that are difficult to modify and where specifically, spirituality could help OA in these conditions to age with greater wellbeing.

## Data Availability

The raw data supporting the conclusions of this article will be made available by the authors, without undue reservation.
